# Significance of Hepatitis B Recurrence in Liver Transplantation Recipients

**DOI:** 10.1155/2020/2489526

**Published:** 2020-08-24

**Authors:** Hong-Shiue Chou, Chih-Hsien Cheng, Hao-Chien Hung, Jin-Chiao Lee, Yu-Chao Wang, Tsung-Han Wu, Chen-Fang Lee, Ting-Jung Wu, Kun-Ming Chan, Wei-Chen Lee

**Affiliations:** ^1^Division of Liver and Transplantation Surgery, Department of General Surgery, Chang-Gung Memorial Hospital, Linkou, Taiwan; ^2^Chang-Gung University College of Medicine, Taoyuan, Taiwan

## Abstract

**Background:**

A combination of antihepatitis B immunoglobulin and antiviral agents is the most common regimen for prophylaxis of hepatitis B recurrence after liver transplantation. However, hepatitis B recurrence still happens. The significance of hepatitis B recurrence is less mentioned.

**Materials:**

Forty-eight of the 313 hepatitis B liver transplant recipients having hepatitis B recurrence were included in this study. The patients were divided into group A, the patients transplanted for hepatitis B-related liver failure, and group B, the patients transplanted for hepatitis B-related cirrhosis and HCC. The clinical manifestations after hepatitis B recurrence were recorded.

**Results:**

Among the 48 patients with hepatitis B recurrence, 23 patients were in group A and 25 patients in group B. The age was 51.6 ± 9.4 years in group A and 52.8 ± 6.4 in group B (*p* = 0.869). The MELD score prior to transplantation was 23.1 ± 9.9 in group A patients and 12.9 ± 5.6 in group B patients (*p* < 0.001). The median (interquartile) interval from transplantation to hepatitis B recurrence was 10 (2-19) months for group A patients and 13 (8.5-35) months for group B patients (*p* = 0.051). After hepatitis B recurrence, the liver function was almost normal in both groups. In group B patients, 10 patients had HCC recurrence with 7 of 10 patients having hepatitis B recurrence earlier than HCC recurrence. The interval between hepatitis B and HCC recurrence was 1 to 15 months. The 1-, 3-, and 5-year survival rates were 82.6%, 73.9%, and 69.0%, respectively, for group A patients and 96%, 76%, and 68%, respectively, for group B patients (*p* = 0.713).

**Conclusion:**

The patients have uneventful liver function under antiviral agent while hepatitis B recurred. For the patients having HCC prior to transplantation, close monitoring of HCC recurrence is necessary if hepatitis B recurs.

## 1. Introduction

Hepatitis B-related liver diseases remain the major indication of liver transplantation in Asia [1]. These hepatitis B-related diseases include acute hepatitis B with liver failure, end stage of hepatitis B-related cirrhosis, and hepatitis B-associated hepatocellular carcinoma (HCC) [2–5]. In the era without prophylaxis of hepatitis B recurrence, hepatitis B would recur under immunosuppression and the clinical course after hepatitis B recurrence was similar to that of fulminant hepatitis B. The liver grafts would fail quickly again. Consequently, hepatitis B-related liver diseases were relatively contraindicated to have liver transplantation.

In the era of prophylaxis of hepatitis B recurrence with the combination of antihepatitis B immunoglobulin and antiviral agents, the clinical course after hepatitis B recurrence is totally different. To date, prophylaxis of hepatitis B recurrence is universally performed by antiviral agents or antiviral agents combined with antihepatitis B immunoglobulin after liver transplantation [6–8]. The outcomes of liver transplantation for hepatitis B-related diseases are even better than other indications of liver transplantation [9]. However, even if hepatitis B recurrence can be effectively prevented by the combination of antihepatitis B immunoglobulin and antiviral agents, around 10-15% of hepatitis B patients still have hepatitis B recurrence after liver transplantation [10, 11]. The clinical course after hepatitis B recurrence is less mentioned.

In this study, we collected the clinical data of the patients with hepatitis B recurrence, who were under the regimen of the combination of low-dose antihepatitis B immunoglobulin and antiviral agents for prophylaxis of hepatitis B recurrence, and focused on clinical manifestation after hepatitis B recurrence, particularly on the patients with HCC.

## 2. Patients and Methods

### 2.1. Patients

313 hepatitis B patients had liver transplantation for hepatitis B-related liver diseases at Chang-Gung Memorial Hospital between 2005 and 2015. All the patients were under the regimen of low-dose antihepatitis B immunoglobulin and antiviral agents for prophylaxis of hepatitis B recurrence. Under the prophylaxis regimen, 48 patients had hepatitis B recurrence. These patients were further divided into two groups: group A, the patients who had liver transplantation for acute or chronic hepatitis B-related liver failure, and group B, the patients who had liver transplantation for hepatitis B-related cirrhosis combined with HCC. The clinical profiles and liver function after hepatitis B recurrence were recorded. The study was approved by the local Ethic Committee of Chang-Gung Memorial Hospital (IBR No. 201701232B0).

### 2.2. Definition of Hepatitis B Recurrence

Surface antigen of hepatitis B (HBs Ag) was measured every 3 months after transplantation. Recurrence of hepatitis B was defined as the reappearance of HBs Ag by quantitative measurement. Quantitative measurement of HBs Ag was performed using the Elecsys HBs Ag assay (Roche Diagnostics GmbH, Mannheim, Germany), conducted as the instructions of the producers. When HBs Ag reappeared, hepatitis B viral DNA would be measured.

### 2.3. Prophylaxis for Hepatitis B Recurrence

The prophylaxis regimen of hepatitis B recurrence was conducted by the combination of low-dose antihepatitis B immunoglobulin (HBIg) and antiviral nucleotide/nucleoside analogs. Briefly, 10000 IU of HBIg was given intravenously at anhepatic phase followed by 2000 IU of HBIg intravenously every day in the first week after transplantation. 800 IU of HBIg was boosted for the patients with quick decline of HBIg every month for 6 courses [12]. Antihepatitis B viral nucleotide/nucleoside analog was given throughout life. If hepatitis B recurred, antiviral agents were continued to suppress hepatitis B virus replication. If hepatitis B viral DNA was detected, the combination of nucleotide and nucleoside analogs was prescribed to suppress hepatitis B virus replication.

### 2.4. Immunosuppressive Regimen

The immunosuppression was conducted by steroid, tacrolimus, and mycophenolate mofetil. After operation, 200 mg/day methylprednisolone was tapered to 20 mg/day over 5 days and discontinued within 3 months. The administration of tacrolimus was delayed and started orally on postoperative day 2 or 3 while renal function returned. The therapeutic trough levels of tacrolimus were achieved within 7 days after transplantation. Mycophenolate mofetil (MMF; 1 g/day) was given orally until 2-4 weeks after transplantation.

### 2.5. Diagnosis of HCC Recurrence

After liver transplantation, the patients were followed up regularly at an outpatient clinic. Liver function tests, measurement of *α*-fetoprotein, and liver sonography were performed every 3 months. Dynamic computed tomography (CT) of the liver was performed if deemed necessary. Recurrence of hepatocellular carcinoma was defined when dynamic CT detected a tumor with a typical HCC imaging pattern in the liver or extrahepatic tumors, and the date of recurrence was the day that CT was done.

### 2.6. Biostatistics

Unpaired Student's *t*-test was used to analyze continuous variables. Categorical variables were analyzed by either chi-square test or Fisher's exact test. All pairwise multiple comparisons were done by the Holm-Sidak method. The survival was calculated using the Kaplan-Meier method and compared between groups using the log-rank test. The statistical analyses were all performed with SigmaPlot 12.3 software for Windows (Systat Software, Inc., San Jose, CA, USA). *p* < 0.05 was considered statistically significant.

## 3. Results

### 3.1. Patients

From 2005 to 2015, 313 hepatitis B patients had liver transplantation at our institute including 147 for acute or chronic hepatitis B-related liver failure and 166 for hepatitis B-related cirrhosis combined with HCC. In this series, 48 of the 313 patients had hepatitis B recurrence after liver transplantation. Among these 48 patients, 23 (15.6%) patients were in group A and 25 patients (15.1%) in group B. The age and gender between groups A and B were not different. But the patients in group A had higher model for end-stage liver disease (MELD) scores than the patients in group B (23.1 ± 9.9 versus 12.9 ± 5.6, *p* < 0.001). The time to hepatitis B recurrence for the patients in group A had a tendency to be shorter than that for the patients in group B (10 (2-9) months versus 13 (8.5-35) months, *p* = 0.051). The clinical characteristics of the patients and the prescription of antiviral agents are listed in Table 1.

### 3.2. Liver Function after Hepatitis B Recurrence

Among the 48 patients with hepatitis B recurrence, the median (interquartile) level of quantitative HBs Ag was 1.135 (0.925-26.095)IU/mL with a range from 0.07 to 31239 IU/mL. Hepatitis B viral DNA was measured, and only 8 patients (16.7%) had DNA detected. After hepatitis B recurrence, aspartate aminotransferase (AST), alanine aminotransferase (ALT), total bilirubin, and international ratio (INR) of prothrombin time were measured. All the liver functions remained stable, and most of the patients had AST and ALT levels within normal limits. In the last visit at the outpatient clinic, the median (interquartile (IQ)) level of AST was 28 (19-33)U/L and the median (IQ) level of ALT was 19 (14-38)U/L, which were not different from the levels of AST and ALT before hepatitis B recurrence (Table 2).

### 3.3. Complications after Hepatitis B Recurrence

For the 23 patients in group A, 8 patients had complications after hepatitis B recurrence, including 4 biliary complication with infection, 2 pneumonia, one acute alcoholic hepatitis, and one spontaneous bacterial infection due to portal vein stenosis. For the 25 patients in group B, 10 patients had HCC recurrence and one patient had coronary heart disease.

### 3.4. Relationship between Hepatitis B and HCC Recurrence

As hepatitis B viral DNA may be inserted into the genome, HCC cells may express HBs Ag [13]. The sequence of hepatitis B and HCC recurrence is not well-known. In this study, 166 hepatitis B patients had HCC simultaneously. 18 of 166 (10.8%) patients had HCC recurrence after transplantation. For the 18 patients with HCC recurrence, 10 occurred in the 25 patients with hepatitis B recurrence and 8 occurred in 141 patients without hepatitis B recurrence (40% versus 5.7%, *p* < 0.001). In the 10 patients with HCC recurrence, 7 patients had hepatitis B recurrence before HCC recurrence. The interval between hepatitis B and HCC recurrence was 0.5 to 15 months. The other 3 patients had HCC recurrence detected before hepatitis B recurrence.

### 3.5. HCC Recurrence in Hepatitis B Recurrent Patients

For the 25 patients in group B, the pathological pictures showed that 15 patients' HCC tumors were within the Milan criteria, 4 were between the Milan and University of California San Francisco (UCSF) criteria, and 6 were beyond the UCSF criteria. Among the 25 patients, 10 patients had HCC recurrence. Three patients' tumors (3/15, 20%) were within the Milan criteria, 2 (2/4, 50%) were between the Milan and UCSF criteria, and 5 (5/6, 83.3%) were beyond the USCF criteria (*p* = 0.01).

### 3.6. Patient Survival

**Figure 1:**
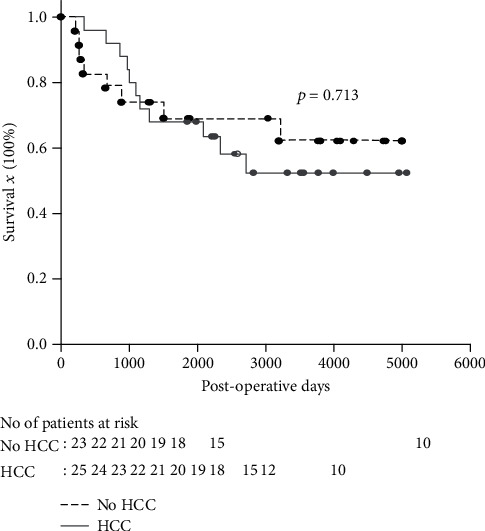
The Kaplan-Meier survival curve of the liver transplant recipients with hepatitis recurrence. The 1-, 3-, and 5-year survival rates were 82.6%, 73.9%, and 69.0% for the patients without HCC and 96.0%, 76.0%, and 68.0% for the patients with HCC prior to liver transplantation.

The median (IQ) follow-up for all 48 patients was 55.7 (32.5-102.5) months with a range from 6.8 months to 144.7 months. The 1-, 3-, and 5-year survival rates were 82.6%, 73.9%, and 69.0% for group A patients and 96.0%, 76.0%, and 68.0% for group B patients, respectively (Figure 1, *p* = 0.713).

## 4. Discussion

Hepatitis B-related cirrhosis and HCC are still the major indications of liver transplantation in Asian countries. Hepatitis B-related liver diseases had been a relative contraindication of liver transplantation until HBIg was introduced to prevent hepatitis B recurrence [14]. Before the HBIg era, hepatitis B might flare up under immunosuppressive agents after liver transplantation. If acute hepatitis B recurred again, liver failure was the fate. After HBIg was introduced to prevent hepatitis B recurrence, graft failure rate was decreased from 80% to 30% [14]. In the middle of 1990s, antihepatitis B nucleoside/nucleotide analogs were introduced for prophylaxis of hepatitis B recurrence [15, 16]. Hepatitis B recurrence could be effectively prevented, and hepatitis B-related diseases were no longer contraindicated. Currently, hepatitis B recurrence can be reduced to around 10% [17–19]. The prognosis of liver transplantation for hepatitis B-related diseases is even better than other benign indications of liver transplantation [9]. The clinical course of hepatitis B recurrence should be reappraised.

Prophylaxis of hepatitis B recurrence is universally carried out by high-/low-dose HBIg and lifelong antiviral nucleotide/nucleoside analogs in most of the centers now [6, 7]. In this study, the hepatitis B recurrent rate was 15.3% by definition. Most of the patients were positive for HBs Ag, but only 16.7% of the patients were positive for hepatitis B viral DNA. Although hepatitis B recurred, the liver function, AST and ALT, and coagulation function were almost normal under continuous antihepatitis B nucleotide/nucleoside analog treatment. Therefore, the current regimen can effectively prevent hepatitis B recurrence. Even if hepatitis B recurs, liver function can still be kept normal with continuous antiviral nucleotide/nucleoside analog treatment. For the patients without HCC prior to liver transplantation, de novo HCC was not found after a median of 55.7 months of follow-up in this study.

The incidence of HCC recurrence is higher in the patients with hepatitis B recurrence than in those without hepatitis B recurrence. In this study, 18 patients had HCC recurrence among 166 patients with liver transplantation for hepatitis B and HCC. Ten patients were among 25 patients with hepatitis B recurrence and 8 patients were among 141 patients without hepatitis B recurrence. Obviously, the reappearance of HBs Ag was associated with a high incidence of HCC recurrence in transplant recipient with hepatitis B and HCC. Although we could not define the HBs Ag coming from hepatocyte or HCC cells, the reappearance of HBs Ag was highly associated HCC recurrence. It is better to be alert for HCC recurrence if HBs Ag reappears.

Obviously, the reappearance of HBs Ag can be recognized as a hint for HCC recurrence surveillance after liver transplantation for hepatitis B with HCC. The relationship between hepatitis B recurrence and HCC recurrence is an interesting issue in liver transplantation. As a fragment of hepatitis B viral DNA may be inserted into the genome of hepatocytes [13], HCC cells also express HBs Ag. While the patients had hepatitis B and HCC, the reappearance of HBs Ag may come from HCC cells and hint the recurrence of HCC. In this series, the reappearance of HBs Ag is earlier than HCC recurrence found in 7 of 10 patients with HCC recurrence. Therefore, for the patients receiving liver transplantation for hepatitis B-associated HCC, the reappearance of HBs Ag hints a high risk of HCC recurrence, particularly for the patients with HCC beyond the Milan criteria.

Infection and HCC recurrence are the major causes of mortality in this study. In this study, the indications of liver transplantation were mainly acute hepatitis B exacerbation with liver failure and end stage of liver cirrhosis for group A patients and HCC for group B patients. Therefore, calculated MELD scores were higher in group A patients than in group B patients. The major complications in group A were infections. Ferrarese et al. in a review article mentioned that cirrhotic patients shared an immunocompromised state which increases the susceptibility to get infections [20]. In group B, the major cause of death was HCC recurrence. Therefore, the first year survival was lower in group A patients than in group B patients. But the 3- and 5-year survival rates were not different.

In conclusion, the patients still have uneventful liver function under antiviral agent control after hepatitis B recurrence. For the patients having liver transplantation for hepatitis B associated with HCC, hepatitis B recurrence is highly associated with HCC recurrence. Close monitoring of HCC recurrence is necessary when HBs Ag reappears.

## Figures and Tables

**Table 1:** The clinical characteristics of the 48 patients with hepatitis B recurrence.

	Group A (*N* = 23)	Group B (*N* = 25)	*p*
Gender (M/F)	19/4	22/3	0.696
Age (years)	51.6 ± 9.4	52.8 ± 6.4	0.869
MELD	23.1 ± 9.9	12.9 ± 5.6	<0.001
Operation			0.106
Deceased donor	9	4	
Living donor	14	21	
Time to recurrence			0.051
Median (IQ)	10 (2-9)	13 (8.5-35)	
Antiviral agents			0.632
Lamivudine	19	18	
Entecavir	3	6	
Tenofovir	1	1	

M: male; F: female; MELD: model for end-stage liver disease; IQ: interquartile.

**Table 2:** Liver function of the 48 patients after hepatitis B recurrence.

	Median	Interquartile	Range	*p*
AST (U/L)				
(b)	25.5	20-44	14-86	0.302
(p)	23	19-33	13-68	
ALT (U/L)				
(b)	29.5	17-57	11-143	0.155
(p)	19	14-38	10-128	
Total bilirubin (mg/dL)				
(b)	1.0	0.6-1.4	0.3-4.7	0.096
(p)	0.8	0.6-1.0	0.2-2.8	
INR of prothrombin				
(b)	1.1.	1-1.1	0.9-1.3	0.073
(p)	1.1	1-1.2	1-1.4	

(b): before hepatitis B recurrence; (p): post hepatitis B recurrence.

## Data Availability

The data used to support the findings of this study are available from the corresponding author upon request.

## Abbreviations

HCC: Hepatocellular carcinoma
HBs Ag: Surface antigen of hepatitis B
HBIg: Antihepatitis B immunoglobulin
HBV: Hepatitis B virus
CT: Computed tomography
MELD: Model for end-stage liver disease
AST: Aspartate aminotransferase
ALT: Alanine aminotransferase
INR: International ratio
IQ: Interquartile
UCSF: University of California San Francisco.
